# Validation of a new diagnostic method for quantification of sleep bruxism activity

**DOI:** 10.1007/s00784-022-04398-w

**Published:** 2022-02-23

**Authors:** Michelle Alicia Ommerborn, Nicole Walentek, Nora Bergmann, Michael Franken, Andreas Gotter, Ralf Schäfer

**Affiliations:** 1 Department of Operative Dentistry, Periodontology, and Endodontology, Faculty of Medicine, Heinrich-Heine-University, Moorenstr. 5, 40225 Düsseldorf, Germany; 2 Clinical Institute of Psychosomatic Medicine and Psychotherapy, Faculty of Medicine, Heinrich-Heine-University, Düsseldorf, Germany; 3 Co. Gofficient UG, Aachen, Germany

**Keywords:** Sleep bruxism, Diagnostic tool, Vacuum-formed sheet, ROC curve, Polysomnography, Tooth attrition

## Abstract

**Objectives:**

To validate a new diagnostic method (DIABRUX) for quantifying sleep bruxism (SB) activity using the current gold standard, polysomnography (PSG), as a criterion in an adequate sample size investigation.

**Materials and methods:**

For SB diagnosis, each participant received a two-night ambulatory PSG including audio–video recordings. The 0.5-mm-thick sheet is produced in a thermoforming process. After diagnosis via PSG, each subject wore the diagnostic sheet for five consecutive nights. The resulting total abrasion on the surface was automatically quantified in pixels by a software specially designed for this purpose.

**Results:**

Forty-five participants (10 SB and 35 non-SB subjects) were included. The difference of the mean pixel score between the SB (M = 1,306, SD = 913) and the non-SB group (M = 381, SD = 483; 3.4 times higher for SB) was statistically significant (*p* < 0.001). The receiver operator characteristic (ROC) analysis revealed a value of 507 pixels as the most appropriate cut-off criterion with a sensitivity of 1.0, a specificity to 0.8, and an area under the curve (AUC) of 0.88. The positive and negative predictive value accounted for 0.59 and 1.0.

**Conclusions:**

The present data confirm that the new diagnostic method is valid and user-friendly that may be used for therapeutic evaluation, and for the acquisition of larger sample sizes within sophisticated study designs.

**Clinical relevance:**

The verified properties of the new diagnostic method allow estimating SB activity before damages occur due to long-standing bruxism activity. Therefore, it might be utilized for preventive dentistry.

**Trial registration number:**

NC T03325920 (September 22, 2017).

## Introduction

Sleep bruxism (SB) has been defined as a masticatory muscle activity during sleep which is characterized as rhythmic (phasic) or non-rhythmic (tonic) and is not a movement disorder or a sleep disorder in otherwise healthy individuals. For the diagnosis of SB, it has been suggested that “possible” SB should be based on a positive self-report, by means of questionnaires and/or the anamnestic part of a clinical examination. “Probable” SB should be based on a positive clinical inspection, with or without a positive self-report, and “definite” SB on positive instrumental assessment, like polysomnography (PSG), with or without a positive self-report or clinical inspection [1].

It is sufficiently known that the PSG in combination with audio–video recordings is characterized by good to very good validity parameters and therefore represents the gold standard for SB diagnosis [2, 3]. However, as discussed elsewhere, the PSG is concomitantly associated with disadvantages such as the technical complexity, time effort, and cost intensity. This limits its use for clinical practice or evaluation of larger sample sizes for scientific purposes, especially in sophisticated study designs with repeated measures [4–8].

Attempts have been made to develop novel electromyographic (EMG) recording systems that offer a more user-friendly technique and better availability compared to the classical PSG [9–11].

A new EMG-independent diagnostic method for quantifying and monitoring of SB activity has been developed for several years ago [12]. It consists of a custom-made diagnostic sheet made of biocompatible material to be worn by the subject similar to a hard plate. The wear caused by rhythmic masticatory muscle activity (RMMA) is automatically quantified by specific software. First results of a pilot study gave an optimistic hint that the new diagnostic method is able to satisfactorily differentiate between SB and non-SB subjects [12]. Moreover, participants rated the new method as simple and user-friendly. However, in this study, SB was assessed using the clinical criteria of the American Academy of Sleep Medicine (AASM) [13].

In order to proof this new diagnostic instrument for its practicability, the next step was to conduct a validation study using the current gold standard, the PSG for the diagnosis of SB, as the validation criterion. Special attention should be paid to the sample characteristics which should be ecologically valid (without too many restrictions, i. e., exclusion criteria that ensure sample homogeneity but prevent clinical representativeness) so that the results can be transferred into clinical practice. Therefore, the objectives of the present study were: (a) to estimate the sensitivity, specificity, and positive and negative predictive values and (b) to evaluate the handling properties of the new diagnostic method for SB quantification and monitoring.

## Materials and methods

### Study design and recruitment of participants

This validation study is a monocentric clinical trial and was performed from January 2018 to August 2020. Forty-five participants took part in the study. Based on the PSG-based diagnostics, the study sample included 10 SB subjects (3 males and 7 females, age range 21 to 46, M = 26.90, SD = 6.97) and 35 non-SB subjects (20 males and 15 females, age range 21 to 40, M = 26.26, SD = 3.55). All subjects gave written informed consent to the procedures approved by the local Ethics Committee (Heinrich-Heine-University of Düsseldorf, registration number 2017094440) and were government-registered as a proof of a medical product trial (EUDAMED No. CIV-17–09-021,645; Clinical Trials.gov ID: NC T03325920). The study complies with the Standards for the Reporting of Diagnostic Accuracy Studies (STARD) statement.

All subjects were native German speakers and were recruited by means of announcements on the homepage of the department and placards on campus. General exclusion criteria were under 20 and over 50 years of age, subjects undergoing current dental or psychological treatment, having more than two missing molars (except third molars), presenting a removable prosthesis or extensive fixed prosthetic restorations, and the presence of gross malocclusion (such as anterior open bite). To exclude serious psychological and psychiatric disorders, each participant completed the Symptom-Check-List 90 (SCL-90-S) [14]. If the calculated Global Severity Index (GSI) as a parameter of general psychological strain exceeded the clinical cut-off (normative reference group), the respective subject had to be excluded. Further exclusion criteria were subjects with pacemakers, pregnant or breastfeeding women, the use of psychotropic drugs, drug and/or alcohol abuse, and the presence of disorders of the central nervous system and/or peripheral nervous system, as well as a lack of knowledge of the German language. Each subject received financial compensation for participation in this time-consuming investigation.

The initial sample size was *n* = 93. After examination of exclusion criteria, *n* = 27 participants were excluded after screening, *n* = 5 were excluded after dental examination, and *n* = 8 participants were excluded after PSG. Exclusion was due to technical problems. Eight participants dropped out on their own during the course of the study.

### Dental examination

As part of the recruitment process, each participant was asked to give a self-assessment concerning the presence or absence of SB indicating “possible” SB [1]. Following a thorough dental examination by one experienced dentist at the Dental Clinic of the Heinrich-Heine-University of Düsseldorf, the presence or absence of “probable” SB was verified: a report of teeth grinding more than five times per week in each week of the preceding 6 months. In addition, SB subjects were required to have at least one of the following symptoms: self-report of orofacial jaw muscle fatigue or tenderness upon awakening, the presence of tooth wear to at least the extend of dentine exposure, and masseter hypertrophy upon voluntary clenching [13, 15, 16]. Non-SB subjects did not show any of these clinical findings. Furthermore, as part of the clinical dental examination, each subject was also scrutinized for signs and symptoms of temporomandibular disorders (TMDs) according to the procedures suggested by the Research Diagnostic Criteria for TMD (RDC/TMD) by the same experienced dentist [17, 18]. Maxillary and mandibular casts were then taken for documentation and to prepare an individual diagnostic sheet for each participant.

### Polysomnographic recordings

The definite diagnosis of SB or non-SB was confirmed by means of ambulatory PSG in the participants’ home environment. Throughout the study, participants and research assistants were blinded to the SB diagnosis. At the end of the study, after completion of all measurements, subjects were informed about their SB diagnosis and corresponding data. Ambulatory full-night sleep recordings were performed on two consecutive nights in the participants’ domestic environment. Recording started automatically 1 h before participants’ preferred bedtime and ended at approximately 9 h or after participants had left bed. Total sleep time had to be at least 5 h. Data were collected using the SOMNOscreen™ digital system (SOMNOmedics, Randersacker, Germany). According to standards, the following non-invasive recordings were performed: 6 electroencephalograms (EEGs; F_1_M_2_, F_2_M_1_, C_1_M_2_, C_2_M_1_, O_1_M_2_, O_2_M_1_), electrocardiogram, bilateral electro-oculograms, bilateral electromyograms (EMGs) of M. tibialis anterior, M. submentalis, oronasal pressure (respiratory airflow), piezoelectric sensors (respiratory effort), pulse oximetry, and body position [19]. Bilateral EMGs for detecting muscle activity of M. masseter and M. temporalis were used. Additionally, audio–video recordings were conducted. Signal quality check and calibration (maximal voluntary contraction for five seconds, voluntary grinding movements, other orofacial movements, e.g. swallowing) were performed on site. EEG signals were recorded and amplified at a sampling rate of 256 Hz and EMG signals with 256 Hz (chin and legs) and 512 Hz (masticatory muscles).

### Analysis of sleep data

Sleep data were analysed and stages scored according to current standards using the SOMNOmedics Diagnostic System (SOMNOmedics GmbH, Germany) [19]. Parameters of interest (total sleep time, sleep efficiency, percentages of stage duration, latencies, duration of wake after sleep onset) were assessed.

### SB diagnosis via PSG

Bruxism episodes were detected and the diagnoses were made based on the criteria of Lavigne and colleagues [2, 16]. RMMA episodes with EMG potentials of at least 20% of maximal voluntary contraction were considered. Episodes were scored as phasic (at least 3 bursts, each lasting 0.25 to 2 s, maximum duration between bursts 3 s), tonic (single burst lasting at least 2 s), or mixed (phasic and tonic RMMA episodes closely related). Facial EMG signals were carefully checked by audio–video recordings for artefacts caused by (orofacial) movements (e.g. swallowing, coughing, sleep talking, stretching, and changing position of body) and for RMMA events during wakefulness. The following indices were calculated and selected as cut-offs for SB diagnosis: (1) number of RMMA episodes/hour of sleep (≥ 4 RMMA episodes/h), (2) number of muscle bursts/hour of sleep (≥ 25 bursts/h), and (3) number of RMMA episodes with grinding sounds (≥ 1 episodes with grinding sounds). Data from the night with the highest number of RMMA episodes/h were included in the analysis.

### New diagnostic method

The newly developed DIABRUX method consists of a 0.50-mm thick sheet of biocompatible Terlux® 2802 HD and a specifically for this purpose developed analysing software [12]. The diagnostic sheet was made up of 5 layers with different colours (first: white, blue, yellow, red, last: green), which allow quantification of the intensity of SB activity. The individual diagnostic sheets were fabricated using pre-existing maxillary stone casts and a thermoforming process, similar to the fabrication of a hard or soft plate. Participants were instructed wearing the diagnostic sheet for 5 consecutive nights, which were independent of the nights of ambulatory PSG. After use, participants returned the worn sheets to the dental clinic with a prepared self-addressed stamped envelope along with a brief handling assessment questionnaire. Subsequently, the worn sheets were digitized under standardized conditions with a reflex camera (Canon 70 D, f-number: f/4, exposure: 1/200 s, ISO 250, focal length: 100 mm). The shooting angle was 90° and the distance to the sheet was fixed. A reference scale on black background provided a defined resolution of 400 dpi. Furthermore, reflections were minimized by using an opaque photo box with indirect illumination from all sides, while colour-neutral light was ensured by LEDs with a colour-rendering index of > 95 [20].

**Fig. 1:**
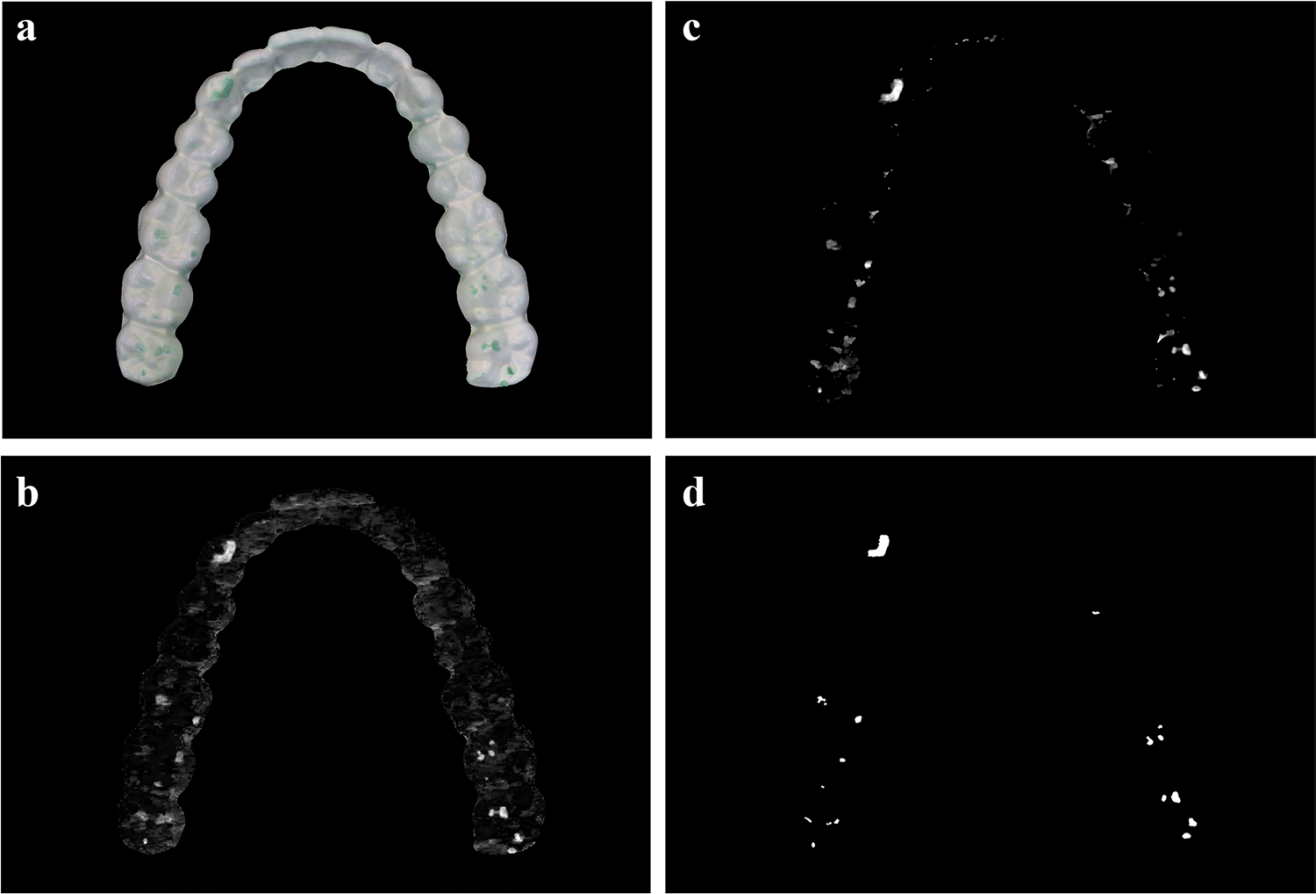
Evaluation of abrasion patterns of the diagnostic sheet using image processing **a** Floating automatic white balance and scrap to detected relevant area (whole sheet). **b** Colour saturation used as first filter for further evaluation. **c** Image after colour selection (here for total abrasion) and median filtering. **d** Black-white image of detected abraded areas after filter application, cut-off and further binary operations

The evaluation itself was performed with an algorithm using image processing and computer vision base algorithms, completed by individual algorithms and linked with an individual macro language (Fig. 1 a–d) [21]. The first step of evaluation was a floating automatic white balance, extracting the shape of the whitened sheet onto black background. Next, a colour saturation filter was defined and applied to an intermediate evaluation layer. The main step of evaluation is a hue mapping and selection. The resulting hue selection map already gives a good hint of where to find the relevant areas. The saturation filter and further specific multiplication, median and binary operations were performed. The same operation steps are repeated for all detected colour layers. In addition, further binary and dilution operations with the detected layer above ensure that no borderline pixel is double counted or missed. Finally, the number of pixels was weighted with a previously empirically determined layer-specific weighting factor depending on the exposed colour respective layer. The combined parameters result in the pixel score.

### Handling properties

To quantify the subjective handling properties of the diagnostic sheet, participants completed a short questionnaire specifically designed for this purpose and previously used in the pilot study [12]. It consists of 5 items and scoring was done by using an 11-point Likert scale: wear comfort of the diagnostic sheet (0 = “very bad” to 10 = “very good”), disturbance (0 = “not at all” to 10 = “very much”), and practicability of the new diagnostic method (0 = “very impractical” to 10 = “very practical”).

### Statistical analysis

Descriptive methods were used to describe the sample. Where necessary, parametric and non-parametric methods (Student’s *t*-tests, *χ*^2^-Test, Wilcoxon rank sum test, and Fisher’s exact test) were used to account for group differences. The alpha error probability was set to *p* = 0.05; correction for multiple comparisons were made by controlling the false discovery rate (FDR) [22]. Two-sided tests were conducted. Normal distribution of data was checked visually via quantile–quantile (QQ) plots and Shapiro–Wilk test.

Receiver operator characteristic (ROC) curve analysis was performed to determine the diagnostic quality of the new diagnostic method. The area under the curve (AUC), sensitivity, specificity, positive predictive value (PPV), negative predictive value (NPV), Youden index, and the corresponding cut-off scores were determined as quality measures. All calculations were made using the data analysing language R [23] and R Studio [24]. ROC analysis was performed using the R Package “ROCit” [25].

## Results

Regarding sociodemographic variables, no significant differences were found between SB and non-SB subjects: sex (χ2(1) = 1.34, *p* = 0.248), age (*W* = 154.00, *p* = 0.571), and education (χ2(1) = 0.24, *p* = 0.627). A similar result was observed for the RDC/TMD diagnoses: there were no significant differences (*p* = 1.00) between the SB (painful TMD, *n* = 2; no painful TMD, *n* = 8) and the non-SB group (painful TMD, *n* = 6; no painful TMD, *n* = 29). Descriptive and statistical group differences concerning SB indices are displayed in Table 1.

**Table 1:** Sleep bruxism indices for SB and non-SB subjects

	SB subjects (*n* = 10)		Non-SB subjects (*n* = 35)			
Variables	Mean (CI 95%)	SD	Mean (CI 95%)	SD	Statistic	*P*^†^
Number of RMMA episodes/hour	8.4 (5.14–11.67)	4.56	2.4 (2.07–2.73)	0.9	350.00^b^	< .001
Number of bursts/hour	57.8 (17.50–98.12)	56.35	8.9 (7.25–10.57)	4.8	350.00^b^	< .001
Number of RMMA episodes with grinding noise^a^	25.0 (− 0.24–50.24)	35.28	1.9 (0.97–2.91)	2.7	295.00^b^	< .001

Standard deviation, *SD*; Confidence interval, *CI*; degrees of freedom, *df*; ^a^Missing values *n* = 2, ^b^Wilcoxon rank sum test, ^†^adjusted *p*-values after controlling the FDR

**Fig. 2:**
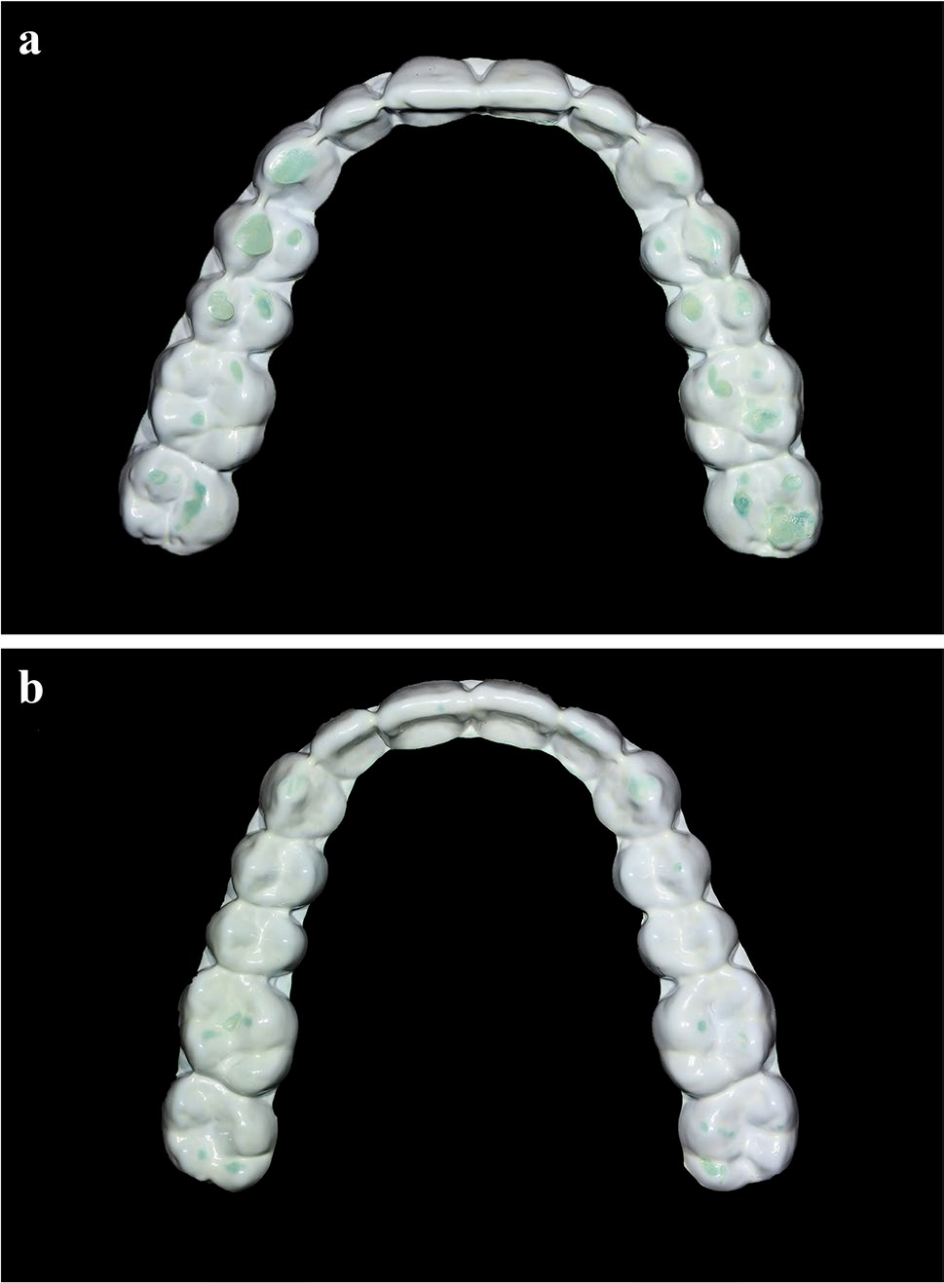
Two different diagnostic sheets, each worn for 5 consecutive nights. **a** displays a sheet from a SB subject (bruxism episodes/hour = 4.40) with pixel score of 1736. **b** shows a sheet from a non-SB subject (bruxism episodes/hour = 1.05) with pixel score of 183

**Fig. 3:**
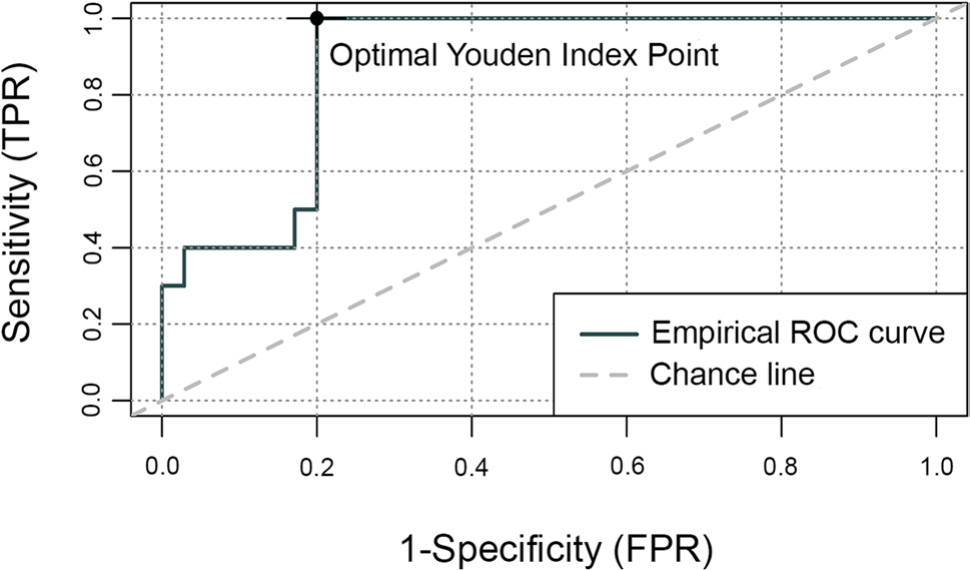
Receiver operator characteristic curve. PSG classification (SB subjects, non-SB subjects) was used as binary outcome. Pixel score of the diagnostic sheet was used as the predictor. Area under the curve (AUC) fits to 0.88. Number of positives = 10, number of negatives = 35. The optimal ratio between sensitivity and specificity (Youden index) is marked by a dot and corresponds to a cut-off pixel score of 507. TPR = true positive rate or sensitivity, FPR = false positive rate or 1 – specificity

The comparison between the SB and non-SB group estimated a mean pixel score of 1306 (SD = 913) for the SB group and a mean pixel score of 381 (SD = 483) for the non-SB group. The obtained difference regarding the mean comparison between the SB and non-SB group, which revealed an approximately 3.4 times higher value in the SB group, was statistically significant (*p* = 0.0002973). This corresponds to an effect size of Hedge’s g (Cohen’s d for unequal group sizes) of 1.54. Accordingly, a post hoc test power of p (1-ß err prob) = 0.98 can be determined. This calculation was performed with the software G*Power [26]. Figure 2 displays two different diagnostic sheets, each worn for 5 consecutive nights. Figure 3 shows the empirically obtained ROC curve using the PSG-based diagnoses (SB vs. non-SB) as binary outcome and pixel score of the diagnostic sheet as predictor. The AUC corresponded to a value of 0.88. After calculating the optimal diagnostic ratio, the cut-off pixel score of 507 was found to be ideal, corresponding to a sensitivity of 100%, a specificity of 80%, a NPV of 100%, and a PPV of 59%. As for the indices of the DIABRUX, significant group differences were observed (Table 2).

**Table 2:** Indices of the new diagnostic method for SB and non-SB subjects

	SB subjects (*n* = 10)		Non-SB subjects (*n* = 35)				
Variable	Mean (CI 95%)	SD	Mean (CI 95%)	SD	Statistic	df	*P*^†^
Pixel score (pixels)	1306 (652–1959)	913	381 (215–547)	483	308.00^a^	-	< .001
Total area of plate (pixels)	221,987 (193,585–250,389)	39,703	240,904 (230,818–250,990)	29,362	− 1.40^b^	11.96	.186
Total abraded area (pixels)	6303 (2979–9626)	4646	1968 (1238–2699)	2126	297.00^a^	-	< .001
Abraded volume (mm^3^)	1.10 (0.58–1.76)	0.80	0.30 (0.19–0.49)	0.40	308.00^a^	-	< .001

^a^Wilcoxon rank sum test, ^b^Student *t* test, ^†^adjusted *p*-values after controlling the FDR

With respect to sleep variables, results are presented in Table 3. Due to multiple comparisons based on nine sleep variables, the *p*-value was corrected after controlling the FDR. There were no significant differences in sleep variables between SB and non-SB subjects.

**Table 3:** Sleep variables for SB and non-SB subjects

	SB subjects (*n* = 10)		Non-SB subjects (*n* = 35)				
Variables	Mean	SD	Mean	SD	Statistic	df	*P*^†^
TST (min)	398.2	46.7	440.5	43.0	− 2.56^a^	13.67	0.206
SE (%)	93.1	4.9	94.6	3.1	155.00^b^	-	0.764
SL (min)	16.7	24.3	7.1	7.4	235.50^b^	-	0.304
RL (min)	74.3	10.5	77.7	29.6	170.50^b^	-	0.913
N1 (%)	5.5	4.2	6.6	4.2	130.50^b^	-	0.517
N2 (%)	41.0	5.3	42.8	5.2	149.00^b^	-	0.739
N3 (%)	26.6	4.4	23.6	5.3	1.85^a^	17.37	0.304
R (%)	26.6	4.2	26.8	4.7	− 0.12^a^	16.08	0.913
WASO (min)	12.6	11.4	15.6	13.5	136.50^b^	-	0.539

^*TST*^, total sleep time; *SE*, sleep efficiency; *SL*, sleep latency; *RL*, REM latency; *WASO*, wake after sleep onset; aStudent’s *t*, ^b^Wilcoxon rank sum test, ^†^adjusted *p*-values after controlling the FDR

**Fig. 4:**
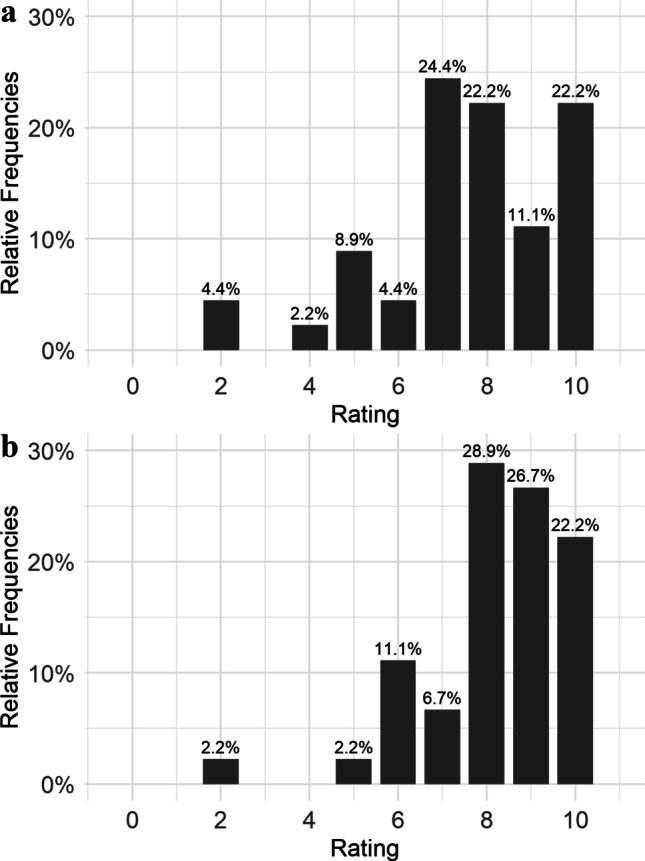
Distribution of subjective ratings of the new diagnostic method. Wear comfort **a** could be rated from 0 = ‘very bad’ to 10 = ‘very good’ and practicability **b** from 0 = ‘very impractical’ to 10 = ‘very practical’

Regarding subjective evaluation of the handling properties of the diagnostic sheet, wear comfort (median = 8; 55% of participant rated 8 or higher) and practicability (median = 8; 78% of participant rated 8 or higher) were rated positively, as higher scores indicate a better rating (Fig. 4a and b). There was no missing data except for the variable number of RMMA episodes with grinding noise (*n* = 2), due to technical problems.

## Discussion

In developing this new diagnostic method, it was of major importance to estimate the SB activity acting on its effector organ, namely the stomatognathic system. Nowadays, in the dental practice, current SB activity and its accompanied partially noxious forces were usually not detected or recorded. The assessment of having SB is usually made retrospectively based on obvious dental damage, especially general wear. Furthermore, in order to develop a future preventive strategy to omit abnormal attrition, wide acceptance on the part of users and prescribing dentists is mandatory. The present study revealed a good to very good agreement between the new diagnostic method for quantifying SB activity and an ambulatory PSG including audio–video recording in a likewise large sample. Moreover, the estimation of the handling properties revealed a strong encouragement by the participants.

The search for a valid and cost-effective diagnostic tool has been and continues to be of interest to researchers, especially since the question of whether SB should be considered pathological and in need of treatment at all cannot be properly answered yet [27]. When comparing the present data with other available diagnostic systems for assessing SB, attention should be paid to the sample size, the type of diagnostic used as criterion, and whether test and reference method have been applied simultaneously or at different measurement times. Keeping in mind the aforementioned, comparisons are hardly possible. For example, a portable device detecting SB episodes by combined surface EMG and heart rate was compared with PSG in 25 subjects, but without synchronized audio–video recording. The application of the PSG electrodes and the Bruxoff device was conducted simultaneously. Indeed, the statistical analysis revealed a very high sensitivity of 92.3% and specificity of 91.6% as well [9]. However, the robustness of these values remains to be proven, as the risk of moderate overestimation in the absence of audio–video recording must be considered. In a pilot study, ten subjects with a clinical diagnosis of SB spent one night in the sleep laboratory. The RMMA index was overestimated by 23.8% without audio–video [28].

Another EMG-based device, the Bitestrip, was also compared with PSG including audio–video recording in a sleep laboratory in 49 SB subjects. The Bitestrip resembles a portable surface EMG device and has a computer chip that registers the number of contractions of the masseteric muscle during the 5-h sleep period for single use. It was placed on the left masseteric muscle simultaneously with the PSG. The results showed an agreement of 87.8%, a sensitivity of 84.2%, positive predictive value of 100%, and a kappa of 0.71 [11]. The authors of this study, including a likewise extensive sample size, concluded from their results that the Bitestrip can be considered a moderate screening method for the diagnosis of SB. However, this study was performed on a sample of patients who all had a history of bruxism resulting in a high positive predictive value due to its association with prevalence (which was obviously very high in this sample). Furthermore, the Bitestrip was used in parallel with the simultaneous PSG and EMG measurement, which must inevitably lead to a high correlation between the measurement methods.

Moreover, a novel quantitative method for tooth grinding surface assessment using 3D scanning has been applied to a single-colour sheet. To simulate tooth grinding, the surface was prepared using a hand piece. The analysis of 18 standardized splints, each with eight grinding surfaces, revealed an intraclass correlation coefficient of 0.998. The authors concluded from their results that the proposed method was innovative, fast, and cost-effective and would support the initial diagnosis of SB [29]. However, the single-colour sheet applied in the cited study has to date never been validated regarding its capability to diagnose SB. Its use is predominantly intended for evaluating occlusal schemes and visualization of grinding patterns during sleep [30–32]. Since RMMAs have not only been observed in SB subjects, but in also 60% of non-SB subjects [33], it remains to be proven whether it is feasible to differentiate between SB subjects non-SB subjects.

In the present study, ambulatory PSG with bilateral EMGs was performed in 45 subjects on two consecutive nights to record muscle activity due to SB of the masseteric and temporalis muscles. Additionally, audio and video recordings were conducted. The final diagnosis of SB was done according to previously published criteria [2, 13, 16] and independently of the application of the new method, which was realized at a later time. Therefore, the technical and methodological efforts made in this validation study strengthen the value of the data collected.

The present study has some limitations that have to be considered when interpreting data. First, a positive predictive value of 59% was acceptable but not good at all. However, it depends on the PSG criterion (binary outcome) and the cut-off value chosen. The latter was deliberately chosen with the aim of detecting all SB subjects. Moreover, since both the PPV and the NPV depend on the respective sample composition evaluated, it is reasonable to assume that this might be a consequence of the likewise low amount of SB subjects in this study. Therefore, the comparative imbalance regarding the number of SB and non-SB subjects represents the second limitation of this study. In this connection, it also should be noted that the new diagnostic method measures a predominantly grinding movement, which represents a kind of abrasive SB activity, in contrast to an EMG recording, which demonstrates every type of muscle activity as mentioned elsewhere [34]. One might suppose that this issue could result in an under detection of clenching type SB activity. However, the present sensitivity value obtained after plotting the ROC curve underpins the very good accordance between the new diagnostic method and the PSG. Moreover, it should be mentioned that the objective of the new method was to predominantly measure and detect the type of SB activity that causes clinically relevant damages on the effector organ, which is the grinding activity.

In addition to the data obtained on the validity of the new diagnostic sheet, the results of the handling properties estimated by the participants represent another important mark. Since both practicability and wear comfort were rated as good to very good, the new diagnostic procedure will have a high level of acceptance among both the using subjects and the prescribing dentists or scientists. This is particularly important, as future research on SB will involve more naturalistic study designs, e.g. investigating large sample sizes [35, 36]. In summary, the new diagnostic method for quantifying SB activity provides good to very good validity parameters and is characterized by a high wear comfort and practicability by its users.

## Conclusion

Concluding from the former, the new diagnostic method for quantification of SB activity provides good to very good validity parameters and, furthermore, is characterized by a high wear comfort and practicability by its users. In terms of a novel preventive strategy to avoid the development of severe generalized attrition, the new diagnostic method will allow the early identification of SB activity before damages occur because of long-standing SB behavior. Moreover, future studies may also verify the usefulness of the new diagnostic method to prevent fractures and restoration failure in individuals with severe SB activity.
